# Comparison of Gamma-Oryzanol Nanoemulsions Fabricated by Different High Energy Techniques

**DOI:** 10.3390/foods13142256

**Published:** 2024-07-17

**Authors:** Rodrigo Jaime-Báez, Jordi Saldo, Rosalía América González-Soto

**Affiliations:** 1 Departamento de Desarrollo Tecnológico, Centro de Desarrollo de Productos Bióticos (CEPROBI), Instituto Politécnico Nacional (IPN), Yautepec 62730, Mexico; 2 Centre de Innovació, Recerca i Transferència en Tecnologia dels Aliments (CIRTTA), MALTA Consolider Team, Animal and Food Science Department, Facultat de Veterinària, Universitat Autònoma de Barcelona, 08193 Bellaterra, Spain; rsoto@ipn.mx; 3 Centro de Investigación de Alimentos (CIAL), Facultad de Ingeniería, Universidad UTE, Quito 170147, Ecuador

**Keywords:** ultrasonication, microfluidizer, UHPH, nanoemulsion, stability, rheology

## Abstract

Gamma-oryzanol (GO) is a bioactive compound that, due to its biological characteristics, can be added to a food matrix. However, the bioactive compound is difficult to incorporate due to its low solubility and stability. A nanoemulsion allows substances to be packaged in nanometric sizes, improving their bioavailability. In this work, a GO nanoemulsion was developed using high-energy techniques. The methodological process began with the formulation of the coarse emulsion, where the emulsifiers (sodium caseinate and citrus pectin), diluent (rice bran oil), and pH were varied to find the most stable formulation. The coarse emulsion was subjected to four high-energy techniques (conventional homogenization, high-pressure homogenization, ultra-high-pressure homogenization, and ultrasonication) to reduce the droplet size. A physical-stability test, rheological-behavior test, image analysis, and particle-size-and-distribution test were conducted to determine which was the best technique. The formulation with the highest stability (pH 5.3) was composed of 87% water, 6.1% sodium caseinate, 0.6% citrus pectin, 6.1% rice bran oil, and 0.2% GO. The ultrasonic treatment obtains the smallest particle size (30.1 ± 1 nm), and the high-pressure treatment obtains the greatest stability (TSI < 0.3), both at 0 and 7 days of storage. High-energy treatments significantly reduce the droplet size of the emulsion, with important differences between each technique.

## 1. Introduction

Bioactive compounds in food refer to all compounds that are mostly without nutritional value but exert a certain bioactive effect on the human body. These compounds play a crucial role in promoting health and preventing diseases. Some of these bioactive compounds can present problems in solubility and stability, so if they are added directly to a food matrix, or if they are consumed in the form of food supplements or nutraceuticals, their functional effect can be drastically diminished [1,2]. Gamma-oryzanol is a bioactive compound of a lipophilic nature, which has a high antioxidant capacity, hypocholesterolemic activity, and regulatory effect on glucose metabolism. However, it has been reported in clinical trials in human and animal model studies that approximately 80% of the entire compound is recovered in feces, showing that only a small percentage of the compound is absorbed, resulting in poor bioavailability [3].

Nanotechnology in the food industry has been used in an attempt to identify opportunities, discoveries, and applications that can significantly improve the processing of food products. The design of nanodelivery systems allows for the assimilation and improvement of the functionality of compounds or ingredients with biological functions. The term “delivery system” refers to the use of a wide range of structuring, encapsulation, and formulation technologies for the delivery of a functional ingredient [4], which can be added to high-consumption foods. Nanometric delivery systems make it possible to improve the stability and functionality of a compound of interest, controlling its release at specific sites and improving its absorption. These delivery systems can be classified into two groups: liquids and solids. In liquid form, nanoemulsions, nanoliposomes, and nanopolymerosomes can be found. In solid types, lipid nanoparticles, polymeric nanoparticles, and nanocrystals are the most common form [5,6]. Each one provides specific features and functions depending on the objective. However, most of these work on the principle of encapsulation [6].

An emulsion is a colloidal system that can be used as an encapsulation method to improve the bioavailability of a compound of interest. However, as it is a thermodynamically unstable system, it is necessary to reduce the droplet size to improve its stability [7]. High-energy techniques can be a useful tool to reduce the emulsion droplet size. Within the high-energy techniques, we can find conventional homogenization, homogenization with high and ultra-high pressures, and ultrasonication. Homogenization with high pressures in equipment, such as the microfluidizer, has a patented homogenization valve composed of microchannels and fixed geometries, which generate high shear forces, turbulence, and cavitation, using pressures of up to 200 MPa [8]. Ultra-high-pressure homogenization (UHPH) is conducted in a patented device that employs pressure of up to 300 MPa to force the fluid through a piston valve of a variable gap. Adiabatic heating can increase the temperature by 2–3 °C/100 MPa, and mechanical forces convert to heat at 14–18 °C/100 MPa. Rapid cooling is included in the pieces of equipment to ensure sample integrity [9]. Finally, the ultrasonication stabilization mechanism is based on the generation of ultrasonic waves throughout the liquid medium, creating compression and traction stresses, which form microbubbles that contract and expand simultaneously. This contraction and expansion of the bubbles causes a collapse known as acoustic cavitation. Acoustic cavitation causes the rupture of the droplets, reducing their size to nanometric scales [10]. The main difference between these four techniques is the principle of homogenization. In techniques that apply pressure, the geometry, size of the valve, and the amount of pressure used will determine the characteristics of the emulsion (size and stability), while in the ultrasonication technique, the acoustic cavitation acts more locally, reducing particle size more efficiently [11].

However, despite the potential of high-energy techniques being able to reduce the size of emulsion droplets, most of the techniques reported in the development of GO nanoemulsions are low-energy techniques, which have the advantage of using a small amount of energy for the generation of droplets at nanometric scales [12,13,14]. However, the use of these techniques in food systems has two main limitations: (1) the commonly used techniques, such as phase inversion and solvent evaporation, employ solvents that are difficult to remove completely, and, in certain quantities, they can be toxic; (2) these kinds of techniques use synthetic surfactants, which have very specific properties and conditions to adequately stabilize the emulsion, and they are used in large quantities, negatively modifying the sensory characteristics of the food to which the nanoemulsion is added [15,16,17]. Finally, the use of low-energy methods produces a broad range of particle sizes, reported to be between 82–993 nm.

The objective of this work was to develop an emulsion with a nanometric droplet size and high stability. To do this, we treated a coarse emulsion with several high-energy techniques and then characterized and compared them. For this, seven formulations of a coarse emulsion were tested. Once the formulation with the greatest stability was found, it was subjected to four high-energy techniques to characterize, compare, and subsequently estimate which is the best technique to achieve a nanoemulsion.

## 2. Materials and Methods

### 2.1. Coarse Emulsion Preparation

For the coarse emulsion development, sodium caseinate (FUDTECH, Cuaultitlán, México, 90% protein content) and citrus pectin (*Mi granero*, Puebla, México, 60% methylation) were used as emulsifiers, and rice bran oil was used as diluent. Table 1 shows the formulations tested to analyze the components’ effect on the stability of the emulsion. The amounts of emulsifiers were varied, while the diluent and two pH values were tested. At this stage of the study, GO was not added to the emulsions.

The preparation of the emulsion began with the dispersion of the emulsifiers in water. Sodium caseinate was added to 70 mL of deionized water and homogenized at 700 RPM for 20 min. The same process was repeated with citrus pectin in 30 mL of deionized water. The emulsifiers were subsequently stored and refrigerated at 3 °C overnight to ensure complete hydration.

The emulsion was developed by the layer–layer method [18], which consisted of two phases. In the first phase, the rice bran oil was added to the sodium caseinate solution. It was subsequently homogenized with a rotor-stator homogenizer (Polytron, Kinematica, PT 1200, Malterns, Switzerland) at 25,000 RPM for 1 min. In the second phase, the solution with citrus pectin was added and homogenized for 1 min with the same equipment and under the same conditions. In Formulation 7A, the pH adjustment was made and homogenized for another minute. The pH adjustment was made with HCl (Hycel, México).

### 2.2. High-Energy Treatments and Characterization

For this stage of the study, 200 mg of gamma-oryzanol reagent grade were added from the Sigma–Aldrich, St. Louis, MO, USA (CDS021604-1G) brand to the rice bran oil (dispersed and diluent phase). The GO and oil were homogenized at 700 RPM for 10 min to ensure complete dispersion. Then, the coarse emulsion with GO was prepared, as mentioned in Section 2.1. To reduce the particle size, four high-energy techniques were conducted in the formulation with the highest stability according to the turbiscan stability index (TSI).

#### 2.2.1. Conventional Homogenization

The conventional homogenization treatment (CH) was conducted on a benchtop Homolab (FBF Italia, Sala Baganza PR, Italy) in which the coarse emulsion was subjected to a 50 MPa treatment.

#### 2.2.2. Ultrasonication

The ultrasonication treatment (US) was conducted with Ultrasonicator equipment (Hielscher, UP200St, Teltow, Germany) using the S26d7 probe at a 90% amplitude and 185 W of power for 3 min. The treated emulsions were placed in an ice-cooling bath.

#### 2.2.3. High-Pressure Homogenization

The high-pressure treatment was conducted in the Microfluidizer M-110L homogenizer (Microfluidics, Westwood, MA, USA), in which two treatments were conducted at 100 MPa pressure. In the first treatment, the emulsion was passed through the equipment for one cycle (HP1). For the second treatment, the emulsion was passed through the equipment for two cycles (HP2).

#### 2.2.4. Ultra-High-Pressure Homogenization

The ultra-high-pressure treatments were processed in a Ypsicon Model A-60 continuous high-pressure system device (Ypsicon Advance Technologies, S.L., Barcelona, Spain). Two treatments were conducted in the device. In the first treatment, the coarse emulsion was subjected to a pressure of 100 MPa (UH100). For Treatment 2, the pressure increased to 200 MPa (UH200).

### 2.3. Physical Stability of Untreated and High Energy Treated Emulsions

The physical stability of the emulsion with and without high-energy treatments was determined using the Turbiscan LAB optical analyzer equipment (Formulaction, Toulouse, France). The equipment detects the change in particle size in destabilization processes such as coalescence, flocculation, and phase separation. Stability is measured by the light intensity in transmission (T) and backscattering (BS). For this process, the equipment has an optical head with an infrared light source (λ_air_ = 850 nm) that emits an amount of energy in the form of photons through the solution to be analyzed and two detectors (T and BS). The incident energy passes through the sample and is detected by the detector T. This energy is known as transmitted energy. The energy that fails to pass through the sample is called backscatter energy, or BS, and is detected by the BS detector.

The overall stability of the sample is detected by the equipment using the stability index (Turbiscan Stability index (TSI)), which is represented by the following equation [19]:

(1) $$TSI=\sum_{i}\frac{\sum_{n}\left|scan_{i}\left(h\right)-scan_{i-1}\left(h\right)\right|}{H}$$

where:

$scan_{i}\left(h\right)$: Backscatter measurement at a particular height

*H*: total height of the sample, from the bottom to the meniscus

### 2.4. Particle Size and Distribution

The particle size (Z-average size, D10, D50 and D90) and polydispersity index (PDI) were determined by the Zetasizer equipment (Malvern panalytical, Nano ZS90, Malvern, UK). The equipment worked at an angle of 630 nm at 25 °C with a backscatter angle of 173°. A 1:7 dilution of the samples in deionized water was used to determine the particle size and distribution. A triplicate measurement was conducted on the emulsion without high-energy treatment as a control, and the emulsions were treated with high-energy treatments at Day 0 and 7 days of storage.

### 2.5. Rheological Properties

The rheological characterization properties of the emulsions were conducted on the emulsions without treatment and high-energy treatments at Day 0 and Day 7 of storage, respectively. A HAAKE™ RheoStress™ 1 rotating rheometer (Thermo Electron Corporation, Karlsruhe, Germany) with a geometry of concentric cylinders (Z34 DIN 53019/222-1499) and a hollow outer cup or cylinder (Z34 DIN 53019/222-1498) were used at a temperature of 20 °C. The rheological properties were determined using a flow curve (shear stress versus shear rate) based on the increase and subsequent decrease in the shear rate of 0.001 s⁻¹ and 100 s⁻¹ for 1 min. The flow curve was fitted to the Herschel–Bulkley rheological model, which is expressed by the following equation [20]:

(2) $$\tau =\tau_{0}+k\cdot {\dot{\gamma }}^{n}$$

where:

***τ*** Shear stress (Pa)

***k*** Consistency index (Pa·s^n^)

***τ*_0_** Creep threshold (Pa)

$\dot{\gamma }$ Shear rate (1/s)

***n*** Flow-behavior index

### 2.6. Image and Morphology Analysis: Confocal Laser Scanning Microscopy

The morphology of the emulsion with and without the high-energy treatments was observed on a Confocal Laser Scanning Microscope (CLSM) (Carl Zeiss, LSM800, Jena, Germany). The sample was processed in Zeiss Efficient Navigation (ZEN) Software Version 2.6 Blue Edition. The sample was mounted on a glass slide with a 40X apochromatic objective and numerical aperture of 1.3 (dimensionless value). Autofluorescence was identified using the “Lambda mode” tool, which consisted of a wavelength scan from 300 nm (ultraviolet) to 800 nm (infrared) to detect “autofluorescence” signals, a 488 nm laser, and another 561 nm with an excitation of 4.5%. Finally, we worked with a pinhole aperture of 1 AU (Airy Unit), which was equivalent to 36 µm. The team acquired micrographs, and these were stored in TIFF format with a resolution of 1024 × 1024 pixels.

### 2.7. Statistical Analysis

In the physical-stability analysis, three replicates for particle size and distribution were conducted. A two-way ANOVA was performed on particle size and distribution, with time and treatment as factors (Tukey’s test, *p* < 0.05). The statistical software used was SigmaPlot (Version 12.0).

## 3. Results and Discussion

### 3.1. Coarse Emulsion Physical Stability

**Figure 1:**
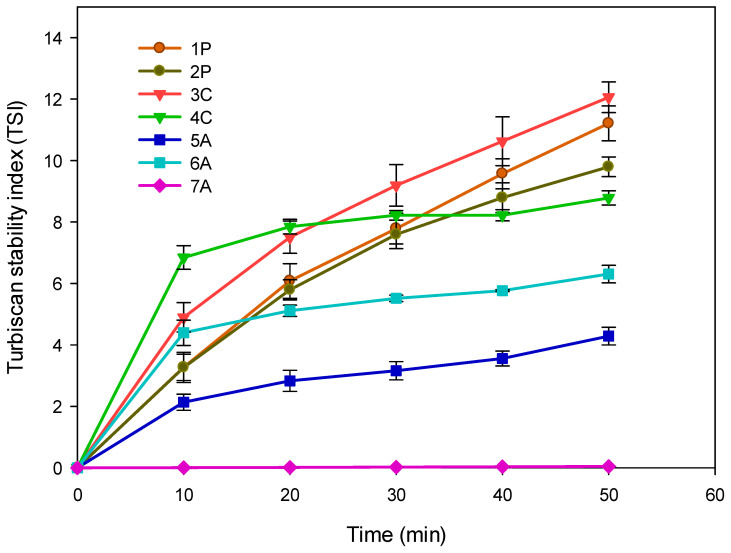
Turbiscan stability index in coarse emulsion formulations: 1P (0.3 g pectin), 2P (0.7 g pectin), 3C (5 g sodium caseinate), 4C (7 g sodium caseinate), 5A (7 g oil), 6A (9 g oil), and 7A (pH 5.3).

The physical stability of the coarse emulsions by the TSI stability index is shown in Figure 1. The changes in the TSI show the effect on the components of the seven formulations tested. In the 1P and 2P formulations, the amount of pectin was varied, keeping the amount of sodium caseinate and rice bran oil constant. The stability index shows a lower value when the amount of pectin is increased (0.7 g). In formulations 3C and 4C, two amounts of sodium caseinate were tested, with the amount of pectin and rice bran oil kept constant. In this formulation, the greatest amount of sodium caseinate (7 g) shows better stability. For Formulations 5A and 6A, the amount of oil was varied, keeping constant the amount of emulsifiers. In this formulation, the amount of emulsifier (stabilizing material) and oil was important to achieve longer stability times for both formulations. In Formulation 6A, the emulsifying material (7.7 g) is less than the oil (9 g), showing a higher value compared to Formulation 5A (7 g). This is also comparable with the previous formulations. In the 1P, 2P, and 3C formulations, the amount of oil was greater than the amount of emulsifier. On the other hand, in the 4C formulation in which the amount of emulsifier is greater than that of the oil, saturation occurs in the emulsified system, which in the long term can provide instability in the entire emulsified system [21]. Formulation 7A with the pH adjustment had the lowest index value of all formulations tested.

The pH effect on emulsifiers is due to their chemical characteristics. Sodium caseinate, due to its amphiphilic nature, tends to be located at the interface of the oil droplet and the aqueous phase. On the other hand, citrus pectin, which has hydrophilic characteristics, will be located in the continuous phase (water) of the emulsion. However, both sodium caseinate and citrus pectin have an electronegative charge, so in the same formulation, they tend to repel each other, preventing the interaction between them [22]. An adjustment in the pH of the emulsion, close to the isoelectric point of the protein (<5.5), produces protonation, changing the electrical profile of the polysaccharide to positive, promoting an association between the two biopolymers [18,22]. This is why Formulation 7A had the lowest values in the TSI and the highest stability in the backscattering profile. For these results, Formulation 7A, which has the highest stability, was treated with the four high-energy techniques.

### 3.2. Particle Size and Distribution

The particle size and PDI of the emulsions with and without high-energy treatments on Days 0 and 7 of storage are shown in Table 2. The particle-size results show statistically significant differences between the emulsions with and without high-energy treatments. All high-energy treatments showed significant statistical differences between each other, and no significant differences were observed between the two storage times for the same treatment. The PDI value shows an increase on Day 7 for all of the emulsions.

**Figure 2:**
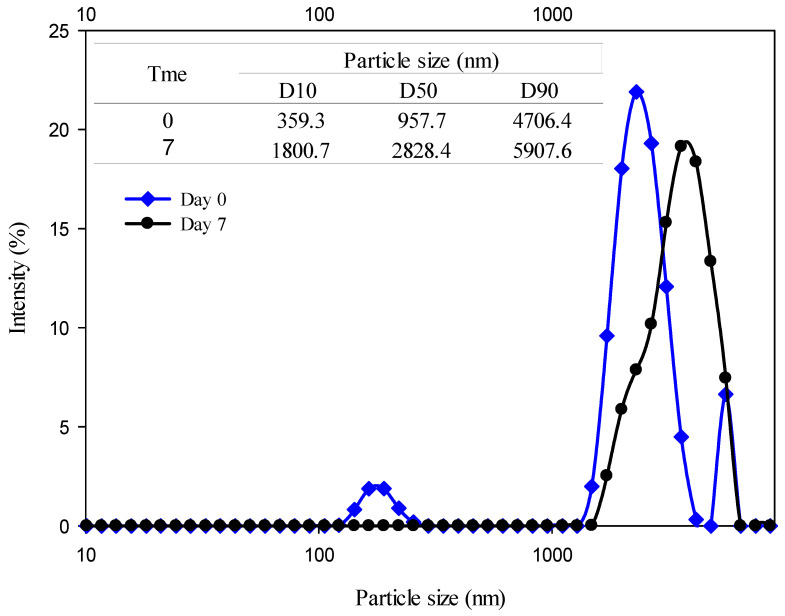
Particle-size distribution for the emulsion without treatment at Day 0 and Day 7 of storage.

The emulsion without treatment was the one that presented the largest particle sizes and PDI values since this emulsion at Day 7 was the one with the largest particle size and more polydispersity. The size distribution and D10, D50, and D90 values of the emulsion without high-energy treatment are presented in Figure 2. At Day 0 of storage, a polydisperse distribution is shown with a small peak below 200 nm, a second peak most prominent above 2000 nm, and a third peak above 5000 nm. For Day 7 of storage, a shift in the size-distribution curve can be observed in which the peaks from Day 0 change to a large peak close to 3500 nm, which corresponds to the increase in particle size, the value of PDI, and the values corresponding to D10, D50, and D90. This increase in value and the shift in the distribution curve could indicate clarification and creaming as emulsion destabilization phenomena.

**Figure 3:**
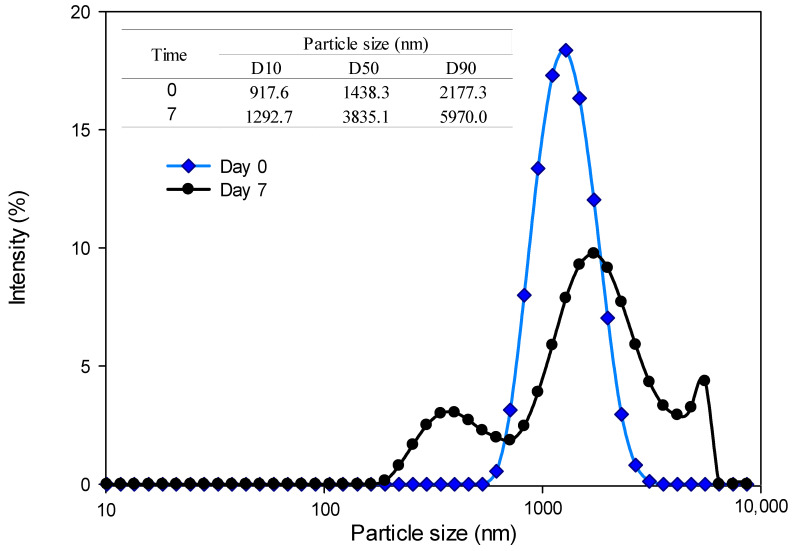
Particle-size distribution for the emulsion with conventional homogenization treatment (CH) at Day 0 and Day 7 of storage.

Among the emulsions subjected to high-energy treatments, the emulsion treated by conventional homogenization (CH) had the largest particle size at both storage times and a polydisperse distribution at Day 7. The size distribution and the D10, D50, and D90 values for this emulsion are shown in Figure 3. A monodisperse distribution can be observed on Day 0 of storage of CH emulsion, which correlates with the low PDI value (0.19) observed in this sample, although the particle size was quite large. A notable change occurred on Day 7 of storage where an increase in the value of the average particle size can be observed. These increases in the Z-average size correspond with PDI and the values of D10, D50, and D90, showing a polydisperse distribution with three peaks: a first peak around 350 nm, a second larger peak around 1500 nm, and a small peak at 5500 nm.

**Figure 4:**
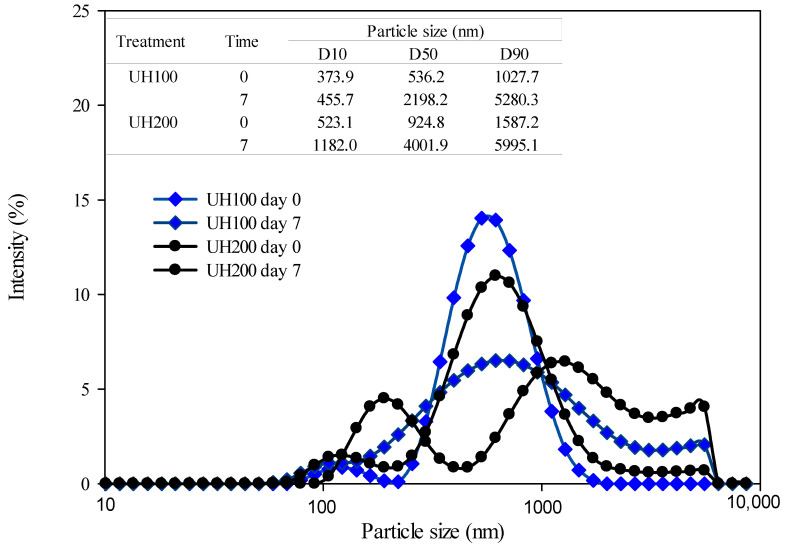
Particle-size distribution for the emulsion with the ultra-high-pressure treatments at 100 MPa (UH100) and 200 MPa (UH200) at Day 0 and Day 7 of storage.

The UH100 and UH200 treatments presented average sizes close to 500 nm, and no statistically significant differences were observed when the pressure and storage-time effect were increased. However, when we look at the particle-size-distribution graph and the values of D10, D50, and D90 (Figure 4), the UH100 sample at Time 0 has a bimodal behavior with a small peak close to 120 nm, and a more prominent peak around 500 nm. This same sample on Day 7 of storage showed an enlargement of the peak with a D50 value of 2198 nm, which corresponds with the increase in the PDI value. On the other hand, the sample subjected to a pressure of 200 MPa (UH200) also presented a bimodal curve at Time 0. However, the values of D10, D50, D90, and PDI were superior to those of the UH100 sample, indicating that although both treatments had a similar average size, the UH200 sample had particle populations with more heterogeneous sizes. By Day 7, both treated emulsions had an increase in particle size and heterogeneity, showing a flatter bimodal curve with an increase in the values of D10, D50, D90, and PDI compared to the UH100 sample at the same storage time.

**Figure 5:**
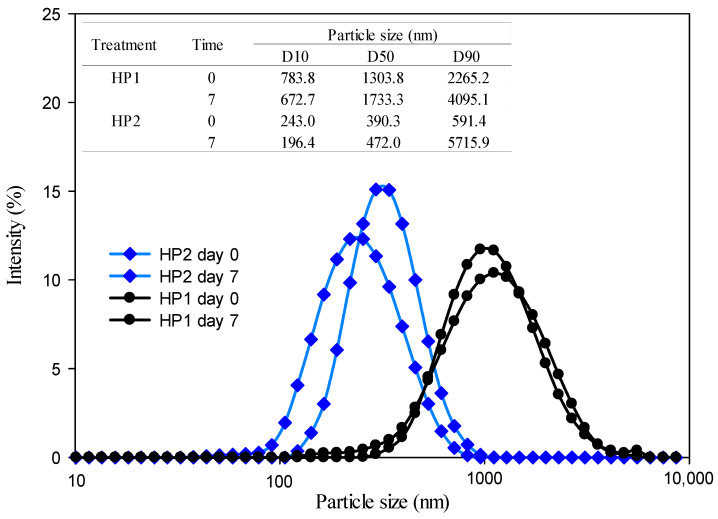
Particle-size distribution for the emulsion with the high-pressure treatments at 100 MPa with one cycle (HP1) and two cycles (HP2) at Day 0 and Day 7 of storage.

In the emulsions treated by high pressure (HP1 and HP2), it can be observed that there is an effect of the number of cycles on the particle size and the PDI. Both values subjected to two cycles decrease in the sample. In addition, emulsions treated by high pressure is the second treatment that presented the smallest particle size at both storage times, as well as the lowest PDI values. For the particle-size distribution, the HP1 and HP2 emulsions presented a monodisperse distribution for both storage times (Figure 5). However, differences can be observed in the values of D10, D50, and D90, showing a decrease in these in the treatment where the emulsion was subjected to two cycles (HP2). This indicates that with this treatment, emulsions with small and homogeneous drop sizes were obtained that were maintained over time, which could be reflected in high stability for these samples.

**Figure 6:**
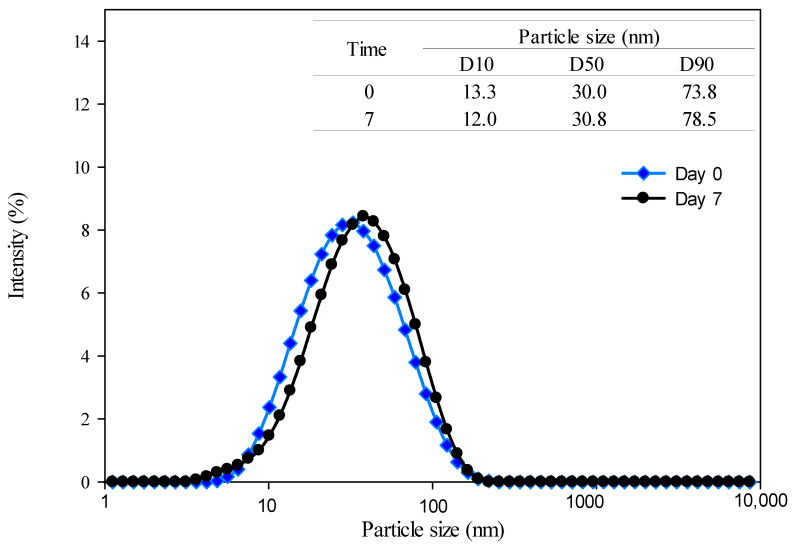
Particle-size distribution of the emulsion with ultrasonication treatment at Day 0 and Day 7 of storage.

The emulsion with the US treatment presented the smallest particle size in both storage times with statistically significant differences in comparison with the emulsion without treatment and the emulsions treated by high-energy techniques. For the particle-size distribution, this emulsion at Day 0 of storage presented a monodisperse distribution and smaller particle sizes in the D10, D50, and D90 values (Figure 6). For Day 7 of storage, the size distribution and the D10, D50, and D90 values remained without changes.

The particle-size values in the emulsions with the high-energy treatments are similar and lower than those reported in the literature in similar emulsified systems with natural and synthetic emulsifiers. Liao et al. [23], in their emulsions treated by homogenization at high pressures using sodium caseinate as an emulsifier, report particle sizes of 147.3–273.1 nm with a PDI of 0.313–0.451. In another study, Liao et al. [24] analyzed the effect of pectin on emulsions stabilized with sodium caseinate at neutral pH, using microfluidization as a high-energy technique, reported sizes from 275–325 nm with a PDI of 0.25–0.35, respectively. Finally, Shamsara et al. [25] developed emulsions based on a combination of pectin and apricot protein, which were treated with ultrasonication at a frequency of 20 kHz and a power of 1300 W. They report sizes between 300–800 nm. For emulsions treated by ultrasonication, Teng et al. [26] reported in their β-carotene emulsions stabilized with soy protein and phosphatidylcholine particle sizes between 291.8 nm and 589.7 nm with a PDI of 0.22 to 0.58. These conditions are like those reported in the present work, with an ultrasonication of 80% amplitude for 6 min. Another study where ultrasonication was used reports sizes > 800 nm with a PDI < 0.2 in their emulsions stabilized with chitosan [27].

In other studies using synthetic surfactants, Sharma et al. [28], in their curcumin emulsions using Tween-20 as a surfactant and ultrasonication as a high-energy technique, reported particle sizes of 143–187 nm with a PDI of 0.181–0.258. Sahafi et al. [29], who developed α-tocopherol emulsions using ultrasonication at 20 kHz with a power of 300 W and a treatment time of 50 min, reported particle size values between 36.65–43.17 nm with a PDI of 0.232–0.240.

For the distribution of sizes and PDI values related to storage time in treatments that involve pressure, some problems related to the use of high and ultra-high pressures are reported in the literature. Fernández–Ávila et al. [10] reported that this is due to the high pressure and temperature reached by the equipment, causing long-term denaturation and aggregation of proteins, producing destabilization phenomena in the emulsifying system. Concerning this, Aghababaei et al. [30] and Varela et al. [31] reported a similar phenomenon in their emulsion treated by ultra-high pressures (100 and 200 MPa), which involves protein aggregation and denaturation. However, they report particle sizes on millimeter scales, a size much larger than what was reported in our study.

In the studies mentioned before [23,24], nanoemulsions stabilized with natural emulsifiers (proteins or polysaccharides) that tend to have a larger particle size compared to those stabilized with synthetic surfactants. This is due to the high molecular weight of natural emulsifiers, causing lower bioavailability due to the larger particle size (>250 nm) [32]. In the present study, the smallest particle size (<50 nm) was obtained using ultrasonication as a high-energy technique and sodium caseinate and citrus pectin as emulsifiers. So far, this is the smallest particle size achieved with this technique using natural emulsifiers.

### 3.3. Rheological Properties

Table 3 shows the results of the rheological properties of the emulsions with and without high-energy treatment on Days 0 and 7 of storage. The results of the flow curves were fitted to the Herschel–Bulkley rheological model, where the equation parameters *k* (consistency index) and *n* (flow behavior index) were taken. The results show a reduction in the *k*-index value in the emulsions treated with high energy. However, differences can be noted between each technique. The US-treated emulsion obtained the lowest value of the *k* index, showing that the ultrasonication treatment could have a greater impact on the viscosity of the emulsion. The emulsions with the HP1, HP2, UH100, and UH200 treatments also managed to reduce the value of the *k* index. However, the ultra-high-pressure treatments (UH100 and UH200) presented higher values. Finally, of the seven treatments, the emulsion with the CH treatment presented the highest value of this index.

For *n* values, the emulsions without treatment and with ultrasonic treatment behaved like a non-Newtonian fluid. However, for the emulsions treated with the use of pressure, they behaved mainly as a Newtonian fluid with a value close to unity, especially in emulsions treated with ultra-high pressures (UH100 and UH200). For the high-pressure treatments (HP1 and HP2) and conventional homogenization treatment, the emulsions also presented an n value not far from one, being the UH100 and UH200 treatments, which are those with a rheological behavior closer to Newtonian. In this sense, Aghababaei et al. [30] and Varela et al. [31] mention that this predominant Newtonian behavior in high-pressure treatments is provided by the formation of protein aggregates that may be responsible for the structuring and constant viscosity of the emulsion. The differences in both high-energy treatments could be due to their mechanism of action, in which ultrasound treatment acts more locally at a molecular level, while for high-pressure treatments, the mechanisms related to high shear, turbulence, and impact affect the viscosity of the emulsified system [33].

The *k* index is a measurement that can be attributable to the total solids content, particle size, and aggregate formation in a suspension [30]. In this case, it can be related to the emulsion components caseinate, pectin, oil, and the difference in drop size depending on the treatment. In this sense, the reduction in the *k* values in the emulsions with the high-energy treatments could be attributed to less friction and a decrease in viscosity because of the reduction in droplet size. In the rheological analysis, the geometry during shearing presents less resistance and greater fluidity, impacting the flow curve and *k* values. This phenomenon was shown in all the emulsions treated with high energy, where the *k* value decreased significantly. This can be explained by the fact that the resistance in the flow curve in the viscosity analysis decreased as an effect of the reduction in the size of the oil droplets [27].

On the other hand, in the emulsions without treatment, an increase in *k* values can be observed, which is possibly attributable to the increase in droplet size and the destabilization of the emulsion.

The flow-behavior and consistency-index values were compared with other authors reported in similar emulsified systems. In the flow behavior, Yang–Yang et al. [33], in their sugar beet pectin emulsions, which were ultrasonicated for 5 and 10 min, report a flow-behavior index of 0.131–0.163. Teixeira et al. [34], in their α-tocopherol emulsion treated with high pressure (1000 bars), report a flow-behavior index very close to unity. In the two studies [33,34], the authors agree that the rheological characteristics of the emulsions will be determined by the surfactants or emulsifiers used, the high-energy treatment used, and the proportion of the continuous phase and the dispersed phase, where the percentage of oil used as the dispersed phase can vary to 5–50%. For the consistency index, Yang–Yang et al. [33] and Teixeira et al. [34] report values of 0.176–0.438 and 0.0022–0.0032 Pa·s^n^, respectively. For these studies [33,34], the authors conclude that the decrease in the consistency index may be indicative of the reduction in the droplet size of the emulsion caused by the high-energy treatment.

### 3.4. Image Analysis of Emulsion With and Without High-Energy Treatment by Confocal Laser Scanning Microscopy

**Figure 7:**
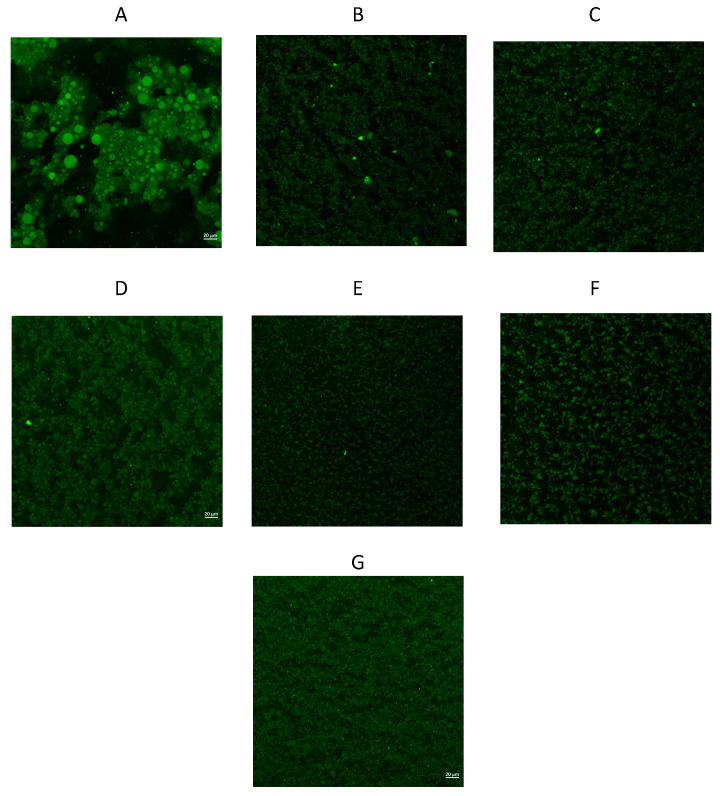
Image analysis by laser scanning confocal microscopy in emulsion without high-energy treatment and emulsions with high-energy treatments: (**A**) emulsion without high-energy treatment, (**B**) emulsion with conventional homogenization treatment, (**C**) emulsion with high-pressure treatment at 100 MPa for 1 cycle, (**D**) emulsion with high-pressure treatment at 100 MPa for two cycles, (**E**) emulsion with ultra-high-pressure treatment al 100 MPa, (**F**) emulsion with ultra-high-pressure treatment at 200 MPa, and (**G**) emulsion with the ultrasonic treatment.

The image analysis of the emulsions without high-energy treatment and emulsions with high-energy treatments are shown in Figure 7 For the emulsion without high-energy treatment (Part A of the figure), it can be noted that the droplet formation throughout the analyzed suspension shows a larger particle size. The most noticeable difference in the emulsions with high-energy treatment with the use of pressure (Figure 7B–F) is the presence of dots with an intense green autofluorescence caused by protein aggregates through all the analyzed suspensions. The presence of aggregates is much more noticeable in the emulsion produced with the conventional homogenization (Part A of the image) and the emulsion with the ultra-high-pressure treatment at 200 MPa (Figure 7F), in which the largest particle size was found. This can be related to the protein aggregates that other authors mentioned [30,31]. For this, Fernández–Ávila et al. [10] mentioned that in emulsions stabilized with protein and treated by high pressures, it is common to observe some protein denaturation causing the formation of aggregates, larger particle sizes, and early destabilization phenomena.

**Figure 8:**
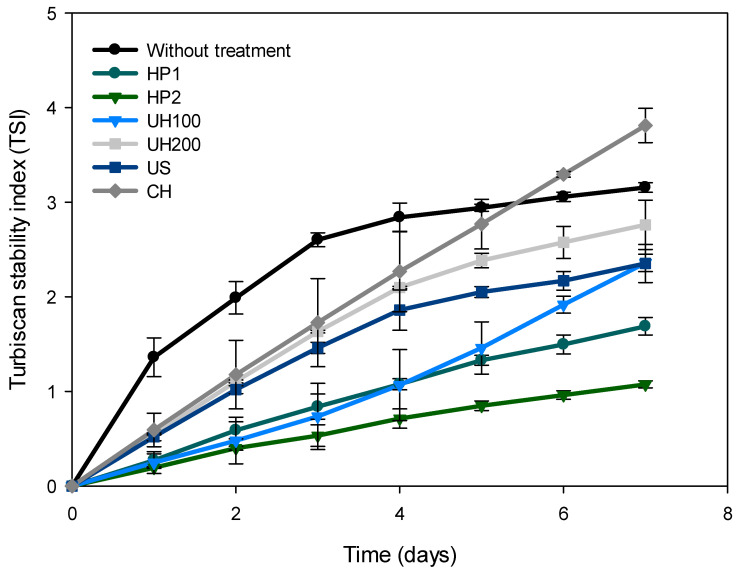
TSI stability index for the emulsion without treatment and the high-energy-treated emulsions: 7A; HP1: High-pressure treatment at 100 MPa for 1 cycle, HP2: High-pressure treatment at 100 MPa for 2 cycles, UH100: Ultra-high-pressure treatment at 100 MPa, UH200: Ultra-high-pressure treatment at 200 MPa, CH: conventional homogenization treatment at 50 MPa and US: ultrasonication at 90% amplitude for 3 min.

The formation of oil droplets shown in the image analysis agrees with that reported by Yue et al. [27], in which the formation of large droplets with high-green fluorescence was observed in the untreated emulsion, for the emulsion treated with ultrasonication, a reduction in the droplet formation is observed, and a red color fluorescence that corresponds to the chitosan, indicating the stabilization of the oil. In this study, the average size reported was around 800 nm. The formation of droplets or aggregates is not observable in the emulsion obtained by the ultrasonication treatment (Figure 8G), as opposed to the images from the other treated emulsions being the smoothest image. This may be because the droplet size of the emulsion is outside the range of the microscope, which is reported to have a limit of 300 nm. The results of the microstructure of the ultrasonic emulsions are consistent with those reported by Salvia–Trujillo et al. [35] where the formation of structures is not noticeable in the analyzed suspension due to its smaller particle size.

### 3.5. Physical Stability of High-Energy Treated Emulsions

The TSI results for high-energy treated emulsions are shown in Figure 8.

The high-pressure treatments (HP1 and HP2) had the highest stability with the lowest TSI value. For these two treatments, high pressure with two cycles (HP2) had the lowest TSI values and the best stability. Treatments that use more energy, such as ultra-high-pressure treatment (UH100 and UH200) and ultrasonic treatment (US), presented higher values in the TSI compared to high-pressure treatment, especially the treatment subjected to ultra-high pressure at 200 MPa (UH200). Finally, the emulsion with the conventional homogenization (CH) treatment had the lowest stability with the highest TSI values, showing that even for Days 6 and 7, the values were higher than the emulsion without treatment.

This reduction in stability in the emulsions with the UH200 and CH treatments could be due to two phenomena: an overprocessing caused by ultra-high pressures that affect the droplet size and emulsification efficiency of the emulsifier, and a lack of energy in the CH treatment to reduce the droplet size. This phenomenon of overprocessing in the ultra-high-pressure treatment could have negatively affected the sodium caseinate used as an emulsifier, of which it has been reported that the use of high pressures denatures and causes aggregates in the protein component that stabilizes the emulsion [30]. This aggregation and denaturation of proteins could initially affect the emulsification efficiency of the protein-type emulsifier and, in the long term, cause destabilization phenomena in the emulsion, which is an undesirable factor that affects the total stability of the emulsion. On the other hand, the droplet size is one of the main factors that affects the stability of the emulsion, causing earlier destabilization phenomena, such as flocculation, if the droplet size is too large and, subsequently, a coalescence phenomenon [30,36].

For emulsion stability, there are four mechanisms that stabilize an emulsion: (1) electrostatic repulsion; (2) steric repulsion; (3) the Marangoni–Gibbs effect; and (4) thin-film stabilization. The electrostatic repulsion is given by the electrostatic force resulting from the interaction between the layers of the emulsifier formed around the oil droplets, which occurs mainly between ionic emulsifiers. Steric stabilization occurs between non-ionic emulsifiers, in which the tail of the emulsifier is located on the outside of the oil drop, which prevents contact between other droplets. The Marangoni–Gibbs effect occurs by absorption of the emulsifier and the formation of an interfacial film, which prevents collision between oil droplets through the formation of a parallel surface. These four mechanisms are related to the characteristics of the surfactant or emulsifier. If this suffers damage in its structure due to high pressure, its emulsifying capacity is reduced [37]. In the results of this work, the reduction in the physical stability of the emulsion treated with ultra-high-pressure techniques could be due to the denaturation and aggregation of sodium caseinate caused by the overprocessing of the high pressure applied, causing a possible negative effect on the steric stability, electrostatic repulsion, and the formation of the stabilizing film, reducing the emulsification efficiency of the emulsifier and its physical stability.

The physical-stability results are compared with those reported by other authors in emulsions treated using high-energy techniques. Aghababaei et al. [30], in their emulsions stabilized with buttermilk (4, 5, 6, and 7%) and treated with ultra-high pressure (100 and 200 MPa), reported TSI values when using 7% buttermilk similar to those obtained in the present work. The amount of emulsifier must be enough to cover the dispersed phase surface. Increasing the ratio of oil stabilizer or reducing the particle size can reach a limit where the emulsion reduces its stability. The lower the value of buttermilk, the higher the TSI value, indicating a less stable emulsion.

For the microfluidizer, Niknam et al. [38,39] prepared nanoemulsions using a response-surface methodology using synthetic surfactants. In both studies, a backscatter profile with low stability is observable if we compare it with our emulsions treated by high pressure through microfluidization and ultrasonication, where a more stable profile can be observed.

For ultrasonic treatment, Teng et al. [26] tested the effect of different ultrasonication operating parameters for the development of β-carotene nanoemulsions using soy protein isolate and phosphatidylcholine. In this, the TSI values (>3) for a treatment like ours (80% amplitude for 6 min) were higher than what was found in our study, showing that the maximum value reported by Teng for his stability analysis was for 6 h. Another study [27] where ultrasonication was used as a treatment with different operation parameters reports TSI values lower than 0.5 in its 5 h analysis for the treatment similar to the present one (40% amplitude for 3 min).

These stability results, which probably involve protein aggregation and denaturation mentioned before, could also be correlated with the results obtained in the image analysis, particle size and distribution, and rheological properties.

In the image analysis, the emulsions with the UH100, UH200, and CH treatments showed the formation of aggregates with high-green fluorescence, with this formation being less evident in the US, HP1, and HP2 treatments. This correlates with the rheological properties, in which the HP1, HP2, and US treatments presented the lowest values in the k index, and the UH100, UH200, and CH treatments presented the highest consistency index, showing an effect in the particle size and distribution. In this, the US treatment obtained the smallest size with statistically significant differences but also presented higher PDI values than the emulsions with the HP1 and HP2 treatments, which obtained larger particle sizes but with a statistically significantly lower PDI value. These results would explain the differences in the final stability of the emulsions with the HP1 and HP2 treatments, being those with the highest stability with lower TSI values and the US treatment with higher TSI values and intermediate stability.

Finally, of all the high-energy treatments, differences can be observed depending on the treatment used, where the high-pressure treatment at 100 MPa for two cycles (HP2) presented the lowest TSI value. However, if we make a comparison with what the other authors found in similar treatments, the stability index at Day 7 in all the treatments in our work is much lower than those reported in the TSI value of fewer than 12 h. This may be because the initial stability of the emulsion without treatment was already high, showing the positive effect of the high-energy treatments on the stability.

## 4. Conclusions

All the high-energy treatments used managed to significantly reduce the particle sizes of all the emulsions, although differences between them can be noted. The emulsions treated with high pressure showed the formation of aggregates, which could be related to the denaturation of proteins, which is an undesirable characteristic that affects the size of the droplets, the physical stability, and the rheological properties. However, the high-pressure treatment at 100 MPa for two cycles was the one that obtained the highest stability. On the other hand, the ultrasonication treatment was the one that produced the smallest particle size with a reduction in the viscosity. This change in the rheological properties is positive, which would facilitate the spray drying in a later stage of stabilizing GO and facilitate the direct incorporation into food products without changes in food texture.

## Figures and Tables

**Table 1:** Formulations evaluated to determine emulsion stability.

Formulation	Citrus Pectin (g)	Sodium Caseinate (g)	Oil (g)	pH
1P	0.3	3	5	7.3
2P	0.7	3	5	7.3
3C	0.7	4	5	7.3
4C	0.7	7	5	7.3
5A	0.7	7	7	7.3
6A	0.7	7	9	7.3
7A	0.7	7	7	5.3

**Table 2:** Particle-size and polydispersity index on the emulsion with and without high-energy treatment.

Emulsion	Storage Time (days)	Z-Average Size (nm)	PDI
Without treatment	0	3497.0 ± 176.9 ^b^	0.40 ± 0.09 ^c^
	7	5290.7 ± 653.4 ^a^	0.65 ± 0.04 ^a^
US	0	30.2 ± 1.0 ^g^	0.26 ± 0.05 ^d^
	7	30.4 ± 1.0 ^g^	0.34 ± 0.05 ^c^
HP1	0	955.0 ± 22.2 ^d^	0.19 ± 0.10 ^e^
	7	928.7 ± 45.2 ^d^	0.27 ± 0.01 ^d^
HP2	0	298.2 ± 9.6 ^f^	0.14 ± 0.05 ^e^
	7	231.1 ± 1.3 ^f^	0.22 ± 0.01 ^d^
UH100	0	481.0 ± 11.5 ^e^	0.29 ± 0.08 ^d^
	7	543.1 ± 8.7 ^e^	0.48 ± 0.01 ^b^
UH200	0	513.3 ± 49.6 ^e^	0.40 ± 0.08 ^c^
	7	692.0 ± 13.9 ^e^	0.63 ± 0.04 ^a^
CH	0	1140.0 ± 17.5 ^c^	0.19 ± 0.04 ^e^
	7	1301.7 ± 44.6 ^c^	0.51 ± 0.05 ^b^

Statistical analysis with two-way ANOVA comparison between two factors (treatment and storage time) with particle size and PDI values (Tukey test *p* < 0.05). Different letters represent significant differences.

**Table 3:** Rheological properties of the emulsion with and without high-energy treatments.

Emulsion	Storage Time (Days)	*k* (mPa.s^n^)	*n*	R^2^
Without treatment	0	180 ± 3 ^c^	0.80 ± 0.02 ^c^	1
	7	860 ± 260 ^a^	0.60 ± 0.07 ^d^	1
US	0	9 ± 2.5 ^g^	0.62 ± 0.03 ^d^	1
	7	4 ± 1.5 ^g^	0.69 ± 0.03 ^e^	1
HP1	0	20 ± 0.5 ^f^	0.86 ± 0.05 ^bc^	1
	7	20 ± 0.6 ^f^	0.72 ± 0.03 ^e^	1
HP2	0	10 ± 0.6 ^g^	0.92 ± 0.03 ^a^	1
	7	10 ± 0.5 ^g^	0.88 ± 0.01 ^a^	1
UH100	0	40 ± 1 ^e^	0.86 ± 0.01 ^bc^	1
	7	40 ± 2.5 ^e^	0.91 ± 0.01 ^a^	1
UH200	0	80 ± 5 ^d^	0.97 ± 0.01 ^a^	1
	7	80 ± 1.8 ^d^	0.96 ± 0.07 ^a^	1
CH	0	170 ± 10 ^c^	0.81 ± 0.01 ^c^	1
	7	530 ± 70 ^b^	0.74 ± 0.03 ^e^	1

The values of *k* and *n* present average, standard deviation values, and the statistical analysis with two-way ANOVA (Factors: treatment and storage time; Tukey test *p* < 0.05). Different letters represent statistical differences (n = 3).

## Data Availability

The original contributions presented in the study are included in the article, further inquiries can be directed to the corresponding author.
